# New products

**DOI:** 10.1155/S1463924687000233

**Published:** 1987


Journal of Automatic Chemistry/*Journal of Clinical Laboratory Automation*, Vol. 9, No. 2 (April–June 1987), pp. 102–104

### New products

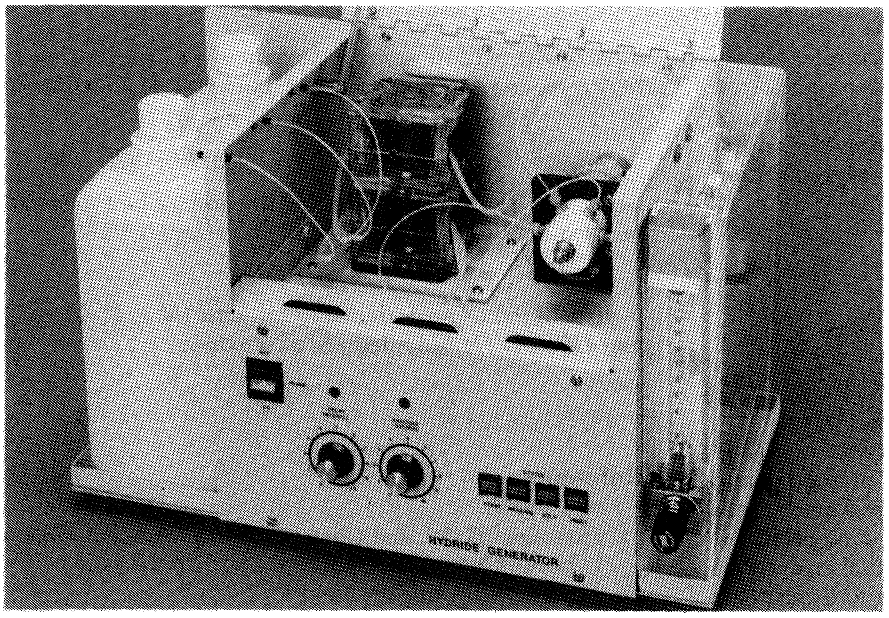

*The new generation PSA hydride generator. Existing customers wishing to upgrade their hydride generators should contact P S Analytical who can provide retrofit kits at extremely attractive prices.*

### Modified pump head enhances performance of PSA hydride generator

The P S Analytical hydride generator is extensively used as a continuous source of hydride generation in ICP, DCP, and atomic absorption spectroscopy. Its inherent design features allow the user of each of these techniques to provide a stable base-line signal with low noise characteristics. In addition, a gas-liquid separator system provides rapid signal rise times and minimal memory effects. In both of these areas, which are fundamental to obtaining excellent detection levels and precision, the pumping action is critical.

Working with Watson Marlow (Falmouth, UK) and using the recently released 304D head arrangement, P S Analytical are now able to offer significant performance advantages over the previous PSA 10.002. The modified pump head is a retrofit addition to existing models. Each head is simply linked to the drive shaft by a twist and snap-in mechanism, no screws are used to fix it to the motor. Correct tensioning in the pump, for the tubing in use, is achieved accurately by adjustment of two sliding clamps in the pump body. Finally, tensioning is added using pair of bayonet-type adjustments. These are released when the pump is not in use, thereby releasing tension in tube and giving prolonged tube life.

Problems experienced with the original pump head: (1) difficulty in adjusting tension correctly; (2) need to dismantle pump head to release tension; or (3) tube welding together and causing a blockage, have all been overcome with this new development.

By avoiding tube deterioration and extending the life of the tube in addition to overcoming the aggravation of dismantling pump head this modification will greatly enhance the attraction of the hydride generator system. Interfaces for the complete range of ICP, DCP and atomic absorption spectrometers are available.

*For further details or for OEM enquiries please contact P S Analytical Ltd, Arthur House, Cray Avenue, Orpington, Kent BR5 3TR, UK. Tel.: 0689 31632.*

### PE 7700 for thermal analysis

Perkin-Elmer's PE 7700 Professional Computer for use with the 7 Series Thermal Analysis System offers improved performance over the PE 7500 in terms of RAM memory and hard disk options. Other benefits include dual floppy disk drive, four R5-232C communications ports, an IEEE-488 communications port and a port for interfacing a streaming tape device for large scale hard disk back-up.

The PE 7700 uses a multitasking/multiuser operating system for direct control of multiple thermal analyser modules which, when combined with one of the several thermal analysis software libraries available, allows simultaneous, independent operation of multiple thermal analysers or data analysis tasks in real time.

*For further information please contact Perkin-Elmer Ltd, Post Office Lane, Beaconsfield, Buckinghamshire HP9 1QA, UK. Tel.: 04946 6161.*

### Ruggedized industrial terminals

Originally designed for use in steel manufacture, a range of rugged terminals is now available from Industrial Peripherals Ltd. The terminals are suitable for use in a variety of applications from harsh shop-floor conditions to laboratories. Made in the UK the 7 in, 9 in and 12 in CRTs are mounted into a metal chassis which is enclosed in a wrap-around steel shroud. This gives excellent protection against physical shock, vibration and impact, as well as dust and splashes.

A detachable sealed sheet keyboard in a metal case is supplied as standard. Moving switch elements under the overlay give a tactile response which is more acceptable to operators than a conventional membrane keyboard.

The internal RS232 interface board gives VT52 compatibility. Comprehensive control codes allow options such as cursor on/off, inverse characters, double size characters and cursor addressing to be selected.

Text mode gives 80 × 25, 64 × 16 or 40 × 16 character formats. The graphics option includes line drawing and pre-programmed symbols which may be used to enhance text displays or for pure block graphics.

Interface standards conform to RS232c and include hardware handshake with CTS, DTR signals or software handshake using the Xon, Xoff protocol. Baud rate and word format may be set using internal switches.

*A full catalogue of industrial computer peripherals is available from: Industrial Peripherals Ltd, Hawarden Industrial Park, Manor Lane, Deeside, Clwyd CH5 3PP, UK. Tel.: 0244 532718.*

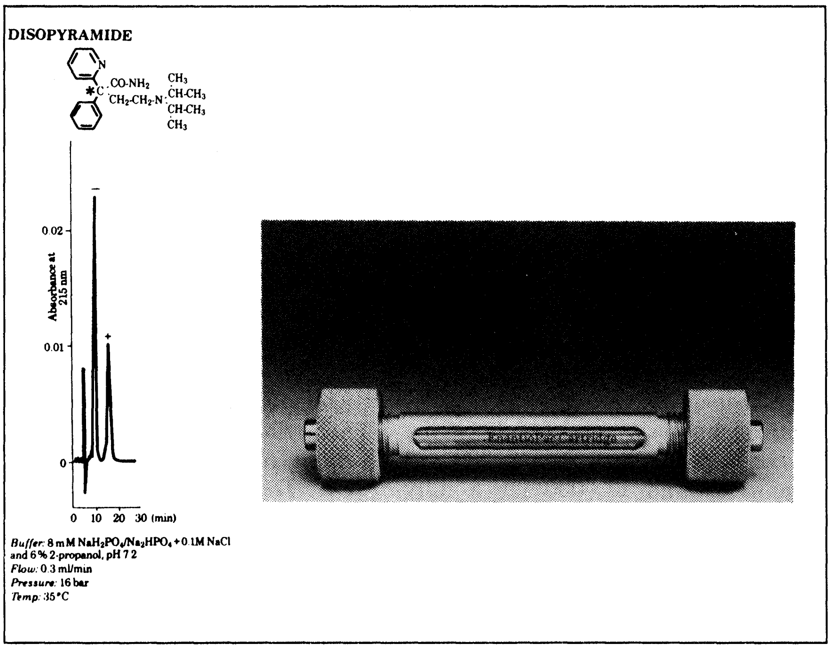

*The EnantioPac cartridge chromatography system.*

### Screening pharmaceuticals for optical purity

More than 50 racemic drugs have now been separated using a new patented HPLC chromatography system. Developed by LKB Produkter of Bromma, Sweden in cooperation with Dr Jörgen Hermansson, the patented EnantioPac cartridge chromatography column system is specifically designed for direct separation and quantitation of optical isomers.

Separations have been made in many different structural families, and include the anti-arrhythmic drug disopyramide antihistamines such as chlorpheniramine, the sedative enhexymal and the local anaesthetic bupivacaine. More than 40 separations have been made using EnantioPac by the FDA.

In contrast to other methods, the EnantioPac chiral column allows separation and quantitation of optical isomers to be carried out without lengthy preparation of derivatives or use of expensive chemical additives. It provides a rapid economical screening process to test optical purity in many different racemic pharmaceuticals. This will help to meet the growing demand to establish the optical purity of new drugs before they can be generally prescribed.

### *Thalidomide*

The cost and difficulty of synthesizing a therapeutic enantiomer in the absence of its partner have resulted in the commercial use of racemic drugs which contains a mixture of the two enantiomers. Normally only one of these is therapeutically active. The second enantiomer may behave as an inactive and harmless contaminant, but it may not. In the case of the sedative drug thalidomide, the non-therapeutic enantiomer triggered abnormal foetal development in pregnant women by damaging growing nerve cells. The tragedy of thalidomide highlights the potential danger of racemic mixtures, and demonstrates the need for an effective screen technique.

The EnantioPac system is able to separate such a wide range of racemic drugs because of the very high number of chiral interaction sites available in the column. The chiral stationary phase comprises a matrix of silica micro-particles (diethylaminoethyl silica, particle size 10 μm) upon which the human plasma protein $\alpha_1$-acid glycoprotein ($\alpha_1$-AGP) has been immobilized. The chemistry of the coupling process allows 10 times as much $\alpha_1$-AGP to be loaded than is possible in conventional chiral columns. Each molecule of $\alpha_1$-AGP provides one chiral interaction site.

When the column is in use, temporary diastereomeric complexes form between the solute enantiomers and the $\alpha_1$-AGP stationary phase. These complexes form at rates determined by their differing stereochemistry, so that the passage of one enantiomer is retarded relative to the other and the separation is made.

### *Cartridge design*

The EnantioPac column comes in cartridge form comprising a reusable holder and pre-packed stainless steel cartridge, measuring 4.0 mm internal diameter × 100 mm long, which (after initial swaging of the steel ferrules) can be fitted by hand. The column can withstand 100 bar pressure and a maximum temperature of 45 °C. It forms part of a complete HPLC set-up including gradient-pump system, auto-injector and variable wavelength monitor for quantitative detection.

Adaptability to varying mobile-phase compositions and other chromatographic conditions such as temperature, pH, organic modifiers and inorganic salts, means that EnantioPac can successfully separate racemic drugs with widely differing hyrophobicity. The system also accepts chiral and non-chiral derivatives of racemates, increasing the number of compounds that can be separated.

*Further information from LKB Produkter AB, Box 305, S-161 26 Bromma, Sweden. Tel.: 46 8 799 8000.*

### Clinical analysis

Beckman have recently added two new instruments to their range of clinical analysis equipment – list prices start at a little over £1300 and rise to around £13 000 for a fully automated version. The System 100 is the lower priced instrument covering virtually the entire spectrum of clinical chemistries with a wavelength range from 340 to 690 nm and reading absorbance values in 0.001A increments for exceptional accuracy. The System 100 also features a built-in filter storage compartment, and quick response optics which read standard 1 cm cuvettes or micro-cuvettes in less than 1 s to provide high throughput. The sealed membrane keyboard is spill-resistant and easy to clean and an automatic one-key zero and blanking facility saves time. No manual calculations for final answers are necessary, and there is a wide selection of optional accessories to extend the capabilities of the system.

The System 700, which will be especially useful in a wide variety of clinical laboratory situations, including large and small health service laboratories, private laboratories and veterinary practices.

The batch analyser accepts average reagent volumes of 600 microlitres and produces up to 200 test results per hour. The system 700 provides 21 pre-programmed Beckman chemistries which can be easily reprogrammed (Beckman has over 50 different reagents for this system) and in addition it features full alpha capability in naming tests and linearity check for kinetic chemistries. An advanced curve fitting programme for Enzyme Immunoassay (EIA) and EMIT is included as standard.

Wavelengths range from 330 to 800 nm with 8 mm bandwidth, using a holographic grating. An advanced flow cell design helps eliminate bubbles, and the cell heats or cools to 25 °C, 30 °C or 37 °C (±0.1 °C). Flow is controlled by a precision pump which steps in 25 μl increments. The system produces carry-over of less than 0.2% at 600 μl. The analyser has an RS232 port for connection to personal computers. Both the 100 and 700 are fully supported by the extensive Beckman range of reagents, standards and controls.

*For further information contact Beckman Ltd, Progress Road, Sands Industrial Estate, High Wycombe, Buckinghamshire, UK. Tel.: 0494 41181.*

### Laboratory Automation Controller

The Zymark Corporation exhibited the new expanded Zymate II Laboratory Automation Controller at the 1987 Pittsburgh Conference in Atlantic City. The Zymate II Controller, with its ability to control up to 25 modules, and to monitor external events is used to program and operate the Zymate Laboratory Robotics System and collect data from other laboratory instruments, as well as to communicate with other computers in the laboratory. The EasyLab software running on the Zymate II Controller can be used directly by laboratory personnel to automate and control laboratory procedures.

The Zymate II Controller is well suited to control laboratory procedures and acquire data necessary for reliable operation and decision making in the laboratory. If large quantities of data are to be processed simultaneously with experiment control, the Zymate II Controller can port data to a laboratory computer. Two interfaces are available to remote computers permitting bidirectional transfer of data and even program control between the Zymate II Controller and the lab computer.

Features highlighted by the manufacturer are:

(1) *String processing capacity*, which allows the Zymate II Controller to receive inputs from the optional laser or wand-type barcode readers and store, print, or pass this information on to another computer.

(2) *Automatic sample scheduling* option, which will convert a user-written procedure to process one sample through a laboratory routine into a procedure which will automatically serialize the routine and schedule the sample flow for simultaneous multiple sample operation.

(3) *The concurrent EasyLab* option allows multiple EasyLab procedures to be running simultaneously. For example, the Controller could be monitoring and controlling modules which are performing an end-point titration while simultaneously weighing out the next sample to be processed.

(4) *On-line user input and promoting* is provided for partially automated or user interactive laboratory procedures.

(5) *Table printing* is included and permits data to be reported on a real-time basis as it is collected or batched and reported at the end of a procedure.

(6) *A remote control/computer interface* option permits the Zymate II to be controlled by a host computer or to simply exchange data with another computer.

(5) *'Plug-in' interfaces for analytical instruments* have also been developed for the Zymate II, allowing instruments to be operated and automatically controlled from the Zymate II Controller. Instruments for which dedicated, preprogrammed interfaces have been developed include: Hewlett-Packard gas chromatographs, liquid chromatographs and UV/Vis spectrophotometers; Mettler balances and titrators; Metrohm titrators and Sartorius balances; Milton Roy and Beckman spectrophotometers.

*More information from Zymark Corporation, Zymark Center, Hopkinton, Massachusetts 01748, USA. Tel.: 617 435 9501.*

